# Transmission of Human Respiratory Syncytial Virus in the Immunocompromised Ferret Model

**DOI:** 10.3390/v10010018

**Published:** 2018-01-02

**Authors:** Leon de Waal, Saskia L. Smits, Edwin J. B. Veldhuis Kroeze, Geert van Amerongen, Marie O. Pohl, Albert D. M. E. Osterhaus, Koert J. Stittelaar

**Affiliations:** 1 Viroclinics Biosciences BV, Rotterdam 3029 AK, The Netherlands; dewaal@viroclinics.com (L.d.W.); smits@viroclinics.com (S.L.S.); edwinvk@gmail.com (E.J.B.V.K.); amerongen@viroclinics.com (G.v.A.); pohl@viroclinics.com (M.O.P.); albert.osterhaus@tiho-hannover.de (A.D.M.E.O.); 2 Department of Viroscience, Erasmus MC, Rotterdam 3015 CN, The Netherlands; 3 Research Centre for Emerging Infections and Zoonoses, University of Veterinary Medicine Hannover, 30559 Hannover, Germany

**Keywords:** respiratory syncytial virus, animal model, ferret, immunocompromised, transmission, contact

## Abstract

Human respiratory syncytial virus (HRSV) causes substantial morbidity and mortality in vulnerable patients, such as the very young, the elderly, and immunocompromised individuals of any age. Nosocomial transmission of HRSV remains a serious challenge in hospital settings, with intervention strategies largely limited to infection control measures, including isolation of cases, high standards of hand hygiene, cohort nursing, and use of personal protective equipment. No vaccines against HRSV are currently available, and treatment options are largely supportive care and expensive monoclonal antibody or antiviral therapy. The limitations of current animal models for HRSV infection impede the development of new preventive and therapeutic agents, and the assessment of their potential for limiting HRSV transmission, in particular in nosocomial settings. Here, we demonstrate the efficient transmission of HRSV from immunocompromised ferrets to both immunocompromised and immunocompetent contact ferrets, with pathological findings reproducing HRSV pathology in humans. The immunocompromised ferret-HRSV model represents a novel tool for the evaluation of intervention strategies against nosocomial transmission of HRSV.

## 1. Introduction

Human respiratory syncytial virus (HRSV) is the leading cause of acute lower respiratory tract infection in children less than 5 years of age, with high hospitalization rates in infants younger than 6 months [1]. It also causes significant morbidity in adults, and contributes to excess mortality in older individuals and immunocompromised individuals of any age [2,3]. In the latter, infection may be prolonged and often complicated by bacterial co-infection, leading to pneumonia and severe respiratory distress [4]. Outbreaks among vulnerable patients in hospital settings are of particular concern [5,6]. Nosocomial transmission poses a substantial risk and remains a serious challenge for hospital infection control. In the absence of an effective vaccine, HRSV treatment options are largely limited to supportive care and expensive monoclonal antibody or antiviral therapy [7,8]. Infection control measures, including isolation of cases, high standards of hand hygiene, cohort nursing, and use of personal protective equipment have been reported as relatively effective in the prevention and control of HRSV outbreaks in nosocomial settings [5,9,10]. The prophylaxis use of monoclonal antibody therapy also can reduce nosocomial transmission [11]. There is nonetheless a need for a larger armamentarium of HRSV-specific treatments and evidence-based control measures for HRSV infection prevention and control.

The lack of an appropriate animal model fully recapitulating HRSV pathogenesis of infection and disease has long represented a hurdle for the development of new intervention strategies against HRSV, including its spread in nosocomial settings [12]. In humans, HRSV is transmitted by direct contact and large aerosol droplets, and via contaminated surfaces, initiating viral replication in the upper respiratory tract after an incubation period of 4–5 days [2]. In severe cases, HRSV further spreads to and replicates in the lower respiratory tract. The virus replicates in ciliated epithelial cells along the respiratory tract, as well as in type I and type II alveolar pneumocytes, resulting in inflammation and necrosis. Animal models for HRSV infection are mostly semi-permissive, requiring a large inoculum of HRSV to result in a productive infection [12]. They include non-human primate (NHP) models, which, apart from chimpanzees and African green monkeys, generally develop no or little clinical signs or pathological changes. The most used non-primate models of HRSV infection are cotton rats, mice, and the neonatal lamb. These models have contributed to improving our understanding of HRSV pathogenesis and host immune responses [13,14]. Furthermore, NHPs and cotton rats may also develop atypical enhanced disease following vaccination with formalin-inactivated HRSV, as seen in humans. However, they all have their limitations [12]. In none of these models HRSV is transmitted to contact individuals, or they are highly impractical to study transmission.

The ferret model of HRSV infection had initially yielded disappointing results, with poor, age-dependent replication of the virus in the lower respiratory tract [12,15]. However, we reported recently that a low-passage clinical isolate of HRSV efficiently replicates in both the upper and lower respiratory tracts of adult ferrets [16]. In addition, we described the use of immunocompromised ferrets as a relevant model for HRSV infection in immunocompromised individuals [16]. It is characterized by high replication levels in the lower respiratory tract, with viral antigen detected in tracheal, bronchial, and bronchiolar ciliated epithelial cells, and by prolonged viral replication and shedding, reflecting the increased viral loads and delayed viral clearance observed in immunocompromised individuals. Prolonged infection is thought to be an important risk factor for nosocomial transmission [17].

Prince et al. reported that infant ferrets generate higher virus loads than adult ferrets, resulting in successful contact transmission of HRSV from infant ferrets to their mother [15]. Recently, Chan et al. showed limited horizontal transmission in a preliminary study between immunocompetent ferrets without proof of replicating virus in the contact ferrets [18]. The immunocompromised ferret-HRSV model [16] offers a new tool to study HRSV transmission and its prevention, which may be used to address the potential of intervention strategies, including vaccines and antibody or antiviral treatments, to block or limit HRSV transmission. Here we report the effective transmission of a low-passage clinical isolate of HRSV from immunocompromised ferrets to both immunocompetent and immunocompromised contact ferrets with the detection of replicating virus in both donor and contact ferrets. Both the intranasal and intratracheal route of infection were explored to determine which site of virus replication would be responsible for transmission of the virus.

## 2. Materials and Methods

### 2.1. Virus

A clinical isolate of HRSV subgroup A strain was isolated in human respiratory epithelium carcinoma (HEp-2) cells from a nasal lavage of an infant hospitalized at Erasmus Medical Centre, Rotterdam, The Netherlands, in 2011. The virus was passaged exclusively in HEp-2 cells, cultured as described previously [16]. The challenge stock used in the animal experiments was passage 3, and was prepared as described previously [16]. Virus titers were determined by end-point titration on monolayers of HEp-2 cells and cytopathic effect (CPE) screening six days after infection. Viral titers were calculated using the method of Spearman and Karber. The challenge stock tested negative for a large panel of respiratory human viruses and mycoplasma by RT-PCR.

### 2.2. Ferrets

Ferrets (*Mustela putorius furo*) were 16- to 18-month-old purpose-bred males, which were seronegative for Aleutian disease virus, and RSV subgroup A and B. They were housed in groups of 2 to 12, as described previously [16]. Male ferrets were selected irrespective of any HRSV-related parameter, because male ferrets would be more robust to endure the treatment procedures. The present experiment complied with all national and international guidelines for care and use of animals and was ethically approved on 15 December 2015 by the Dutch Central Committee Animal Experiments (CCD), protocol number AVD277002015161.

Immunocompromised ferrets received orally twice daily the immunosuppressive medication of 20 mg/kg of body weight (bw) Mycophenolate mofetil (MMF) (CellCept^®^, Roche, Woerden, The Netherlands), 0.5 mg/kg bw tacrolimus (Prograft^®^, Astellas Pharma BV, Leiderdorp, The Netherlands), 8 mg/kg bw prednisolone (Hospital Pharmacy, UMCN St Radboud, Nijmegen, The Netherlands), together with antibiotic prophylaxis of 10 mg/kg bw amoxicillin and 2.5 mg/kg bw clavulanic acid (Pharmachemie BV, Haarlem, The Netherlands), as described previously [16,19].

### 2.3. HRSV Transmission Experiment

**Figure 1:**
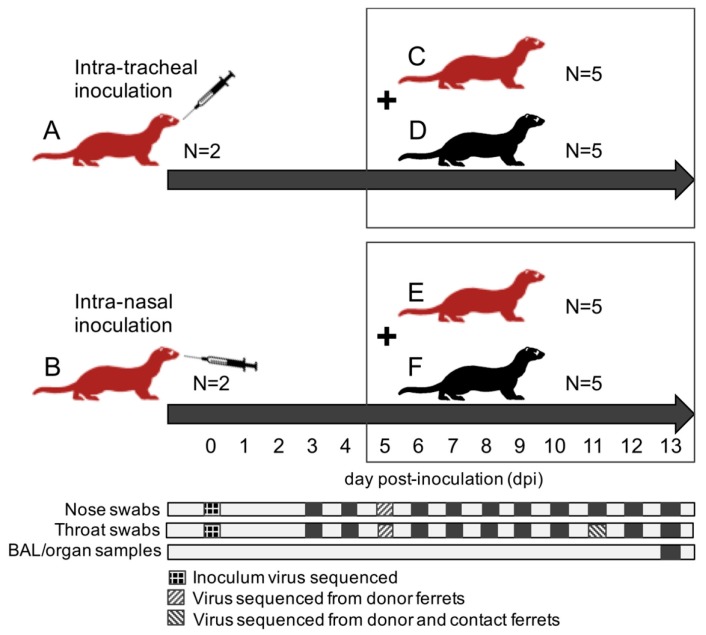
Schematics overview of the experimental design; ferret groups are lettered (**A**–**F**); red ferrets are immunocompromised ferrets; black ferrets are immunocompetent ferrets; contact ferrets (group **C**–**F**) are placed in the same cages as donor ferrets (group **A**,**B**) on day 5 post-inoculation (dpi); all ferrets are euthanized at 13 dpi. Days of collection of nose swabs, throat swabs, broncho-alveolar lavages (BAL), and respiratory tract samples are indicated with dark squares; samples used for viral sequencing are indicated with patterns.

A total of twenty-four ferrets were randomly assigned to the following treatment groups (Figure 1): (A) 2 immunocompromised donor ferrets infected with HRSV via the intra-tracheal route of inoculation; (B) 2 immunocompromised donor ferrets infected with HRSV via the intra-nasal route of inoculation; (C) 5 immunocompromised contact ferrets placed on day 5 post-inoculation (dpi) with group A ferrets; (D) 5 immunocompetent contact ferrets placed on 5 dpi with group A ferrets; (E) 5 immunocompromised contact ferrets placed on 5 dpi with group B ferrets; (F) 5 immunocompetent contact ferrets placed on 5 dpi with group B ferrets.

After a one-week acclimatization period, the ferrets assigned to group A, B, C, and E (Figure 1) were started on the immunosuppression protocol, with daily oral prophylactic antibiotics starting four days before HRSV infection, and twice-daily oral immunosuppressants starting three days before HRSV infection. The ferrets assigned to group A and B were inoculated on day 0. Donor ferrets sedated with a mixture of ketamine and medetomidine (IM doses of 0.2 and 0.01 mL/kg body weight, respectively) were inoculated with 10^5^ median tissue-culture infective dose (TCID_50_) of low-passage clinical isolate HRSV subgroup A by intra-tracheal (IT; group A) or intra-nasal (IN; group B) inoculation with a volume of 3 or 0.3 mL, respectively.

The ferrets assigned to group C to F were introduced in the cages of group A and B ferrets on 5 dpi to enable direct contact. Throat (Copan; rayon tipped) and nose (Copan; polyester tipped) swabs were collected from all ferrets daily from 3 dpi onwards in a 3mL virus transport medium [16]. Donor ferrets were euthanized by exsanguination under anesthesia with ketamine-medetomidine at 13 dpi and contact ferrets at day 8 post-exposure (dpe; corresponding to 13 dpi). Broncho-alveolar lavages (BAL) and samples of the nasal turbinates, trachea, primary bronchus, and lung parenchyma were collected upon autopsy.

All personnel involved in the collection of study data on a day-to-day basis and all personnel performing the laboratory analysis in which interpretation of the data is required were not aware of the so-called Random Treatment Allocation Key at any time prior to completion of the study. All samples were labeled with a unique sample number.

### 2.4. Samples and Assays

All samples were processed within four hours of sample collection. Infectious virus titers and concentrations of viral RNA were measured by virus isolation and reverse transcription-PCR (RT-PCR), respectively, as previously described [16]. Samples of the nasal turbinates, trachea, primary bronchus, and right lung were weighed and subsequently homogenized with a FastPrep-24 (MP Biomedicals, Eindhoven, The Netherlands) in medium and centrifuged briefly before viral load assessment by virus isolation and quantitative RT-PCR.

RNA extraction was performed using a MagNA Pure 96 (Roche Diagnostics Nederland B.V., Almere, The Netherlands) and MagNA Pure 96 DNA and Viral NA Small Volume Kit with an input volume of 190 µL and output volume of 100 µL according to the manufacturer’s instructions (Roche Diagnostics Nederland B.V.). The extraction was internally controlled by the addition of a known concentration of phocine distemper virus (PDV) [20]. 20 µL extracted RNA was amplified in a 50 µL final volume, containing 12.5 µL 4× TaqMan Fast Virus 1-Step Master Mix (Life Technologies, Bleiswijk, The Netherlands), and 1 µL of primers and probe mixture for detection of RSV A, RSV B, and PDV in a triplex reaction (Table S1). The RT-PCR temperature profile was 5 min at 50 °C, 20 sec at 95 °C, 45 cycles of 3 s at 95 °C, and 32 s at 60 °C.

Dilutions of an electron microscopic-counted HRSV stock (ABIOnline.com Columbia) were used for conversion of RT-PCR threshold cycle (*C*t) values into a quantitative measurement of viral particles. Concentrations of viral RNA are expressed as log10 vp/mL. Infectious virus titers in tissue are expressed as log10 TCID_50_ per gram tissue, and infectious virus titer in throat and nose swabs are expressed as log10 TCID_50_/mL. Significant differences in shedding loads were assessed based on the areas under the curve and using the Student *t*-test.

Formalin-fixed tissue sections of nasal turbinates, trachea, and left lung were routinely processed, paraffin embedded, and sectioned at 3–4 μm, deparaffinized with xylene and rehydrated using graded alcohols, and stained with hematoxylin and eosin (H&E) for histopathological examination by light microscopy. For immunohistochemistry (IHC), additional serial slides were sectioned simultaneously and incubated for 1 h with a goat anti-HRSV-peroxidase (PO) (Virostat, Portland, ME, USA) polyclonal antibody following antigen retrieval using citric acid buffer. Endogenous PO was blocked with 3% hydrogen peroxide. The bound PO was visualized by incubating slides with 3-amino-9-ethylcarbazole for 10 min as substrate, resulting in a reddish brown finely-granular staining of HRSV-infected epithelial cells, followed by hematoxylin counterstain. Negative controls were performed in the absence of the antibody.

### 2.5. Nested PCR Amplification and Sanger Sequencing

Full-length F and G gene sequencing was performed on the inoculum virus and RT-PCR positive nose and/or throat swabs collected from the donor ferrets at 5 and 11 dpi and from the contact ferrets at 6 dpe (corresponding to 11 dpi). RNA extraction was performed using a MagNA Pure 96 (Roche Diagnostics) and MagNA Pure 96 DNA and Viral NA Small Volume Kit with an input volume of 200 µL and output volume of 100 µL according to the manufacturer’s instructions (Roche Applied Science). cDNA synthesis was performed using Superscript III Reverse Transcriptase (Invitrogen, Carlsbad, CA, USA). Semi-nested PCR amplification was performed using HotStarTaq DNA Polymerase (Qiagen, Hilden, Germany). Sequence reactions were performed with the BigDye^®^Terminator v3.1 cycle sequencing kit (Applied Biosystems, Foster City, CA, USA) and an ABI Prism 3730 genetic analyzer. Primers are indicated in Table S1.

## 3. Results

### 3.1. Donor Ferrets

**Figure 2:**
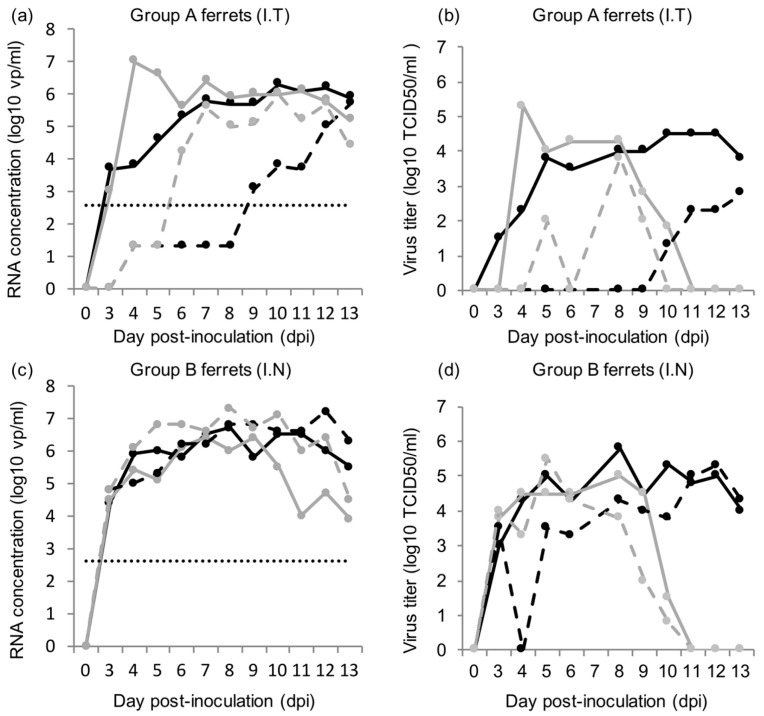
HRSV RNA concentrations and viral titers in the throat and nose swabs of donor ferrets; (**a**) HRSV concentration in the throat swabs (plain line) and nose swabs (dashed line) of group A ferrets inoculated intra-tracheally (I.T); (**b**) HRSV viral titers in the throat swabs (plain line) and nose swabs (dashed line) of group A ferrets inoculated intra-tracheally (I.T); (**c**) HRSV concentration in the throat swabs (plain line) and nose swabs (dashed line) of group B ferrets inoculated intra-nasally (I.N); (**d**) HRSV viral titers in the throat swabs (plain line) and nose swabs (dashed line) of group B ferrets inoculated intra-nasally (I.N); each line represents a single ferret; for (**a**,**c**): quantification limit of qPCR data is indicated by a dotted line, and non-quantifiable *C*t values were assigned the value 1.3 log10 vp/mL.

All donor ferrets inoculated with HRSV either intra-tracheally (group A) or intra-nasally (group B) developed a productive infection. Both HRSV RNA and replication-competent virus were detected in throat and nose swabs of group A and group B ferrets (Figure 2).

**Figure 3:**
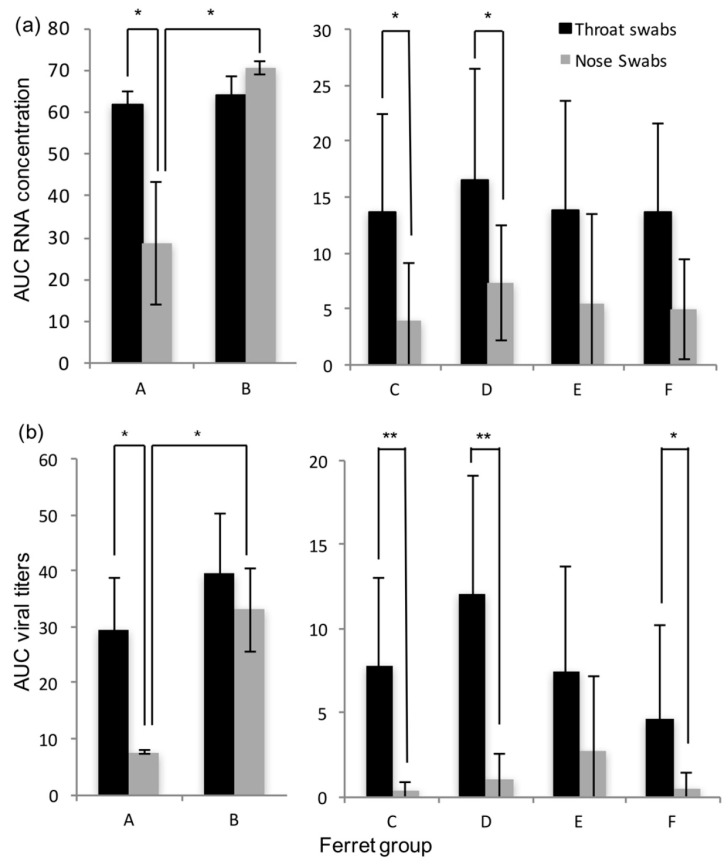
Average RNA concentrations and viral titers in the throat and nose swabs of group A to F ferrets, expressed as areas under the curve (AUC); (**a**) AUC RNA concentration; (**b**) AUC viral titers; significant differences are marked with asterisks (* *p* ≤ 0.05 and ** *p* ≤ 0.01); error bars indicate standard deviations.

Detectable RNA concentrations and viral titers in throat and nose swabs were typically recorded from 3–4 dpi to 10–13 dpi, except in the nose swabs of group A ferrets, with quantifiable RNA concentrations and viral titers appearing later, between 6 and 10 dpi. In group A ferrets, viral RNA concentrations and viral titers were found to be significantly higher in the throat swabs than in the nose swabs, based on the areas under the curve (3.17x, *p* = 0.04 and 3.21x, *p* = 0.04, respectively; Figure 3). Viral RNA concentrations and viral titers in the nose swabs of group A ferrets were significantly lower than in the nose swabs of group B ferrets, based on the areas under the curve (−4.05x, *p* = 0.03 and 4.8x, *p* = 0.02, respectively; Figure 3). HRSV RNA concentrations and viral titers in organ samples and BAL are given in Table 1.

**Figure 4:**
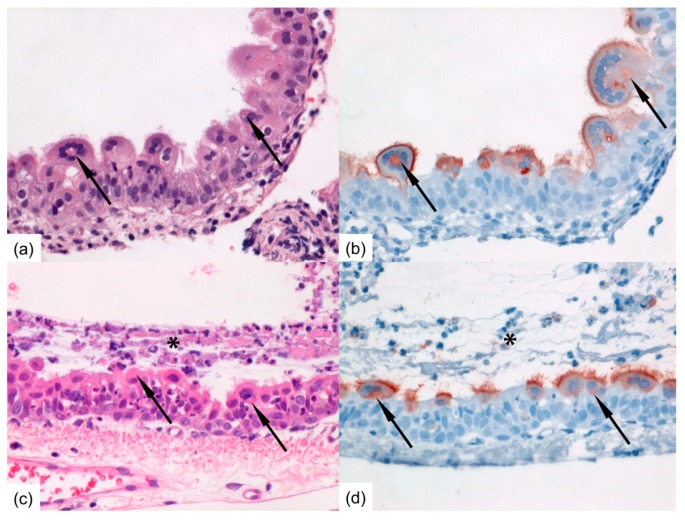
Photomicrographs of intranasally HRSV-inoculated immunocompromised donor ferret (animal #3) inflamed mucosa of the nasal turbinates at 13 dpi (**a**,**b**) and of immunocompromised contact ferret (animal #10) tracheal mucosa following productive HRSV transmission at 8 days post-exposure (**c**,**d**). (**a**) RSV-infected epithelial cells may develop bright eosinophilic viral inclusion bodies (arrows) and may form syncytial multinucleated cells (H&E-stain); (**b**) Serial slide shows HRSV-antigen expression faintly of the cytoplasmic inclusion bodies (arrows) enclosed by a corona of multiple nuclei, and markedly of the outer cellular membranes and cilia of RSV-infected nasal epithelial cells as red-brown staining (Immunoperoxidase, hematoxylin-counterstain); (**c**) The mucosa is markedly inflamed with infiltrated neutrophils and covered with layer of exudate (asterisk); multinucleated epithelial syncytial cells containing bright eosinophilic viral inclusion bodies (arrows) are present within RSV-infected tracheal epithelial cells (H&E-stain); (**d**) Serial slide shows HRSV-antigen expression faintly of the cytoplasmic inclusion bodies (arrows) and markedly of the apical side and cilia of RSV-infected tracheal epithelial cells as red-brown staining (Immunoperoxidase, hematoxylin-counterstain). Original magnifications 400×.

Upon microscopic examination, pathological changes were observed in some of the respiratory tissues sampled from group A and group B ferrets (Figure 4). In each group, cells positive for HRSV-antigen were detected by immunohistochemistry in one of the inoculated ferrets. In the group A positive ferret, positive cells were found in small to moderate number in ciliated epithelial cells of the nasal turbinates, trachea, primary bronchus, and bronchioles. Positive syncytial cells were observed in the nasal turbinates. In the group B positive ferret, positive cells were found in moderate number in ciliated epithelial cells of the primary bronchus and in large number in ciliated epithelial cells of the nasal turbinates and trachea, including positive syncytial cells (Figure 4).

### 3.2. Contact Ferrets

HRSV was detected by RT-PCR in the throat swabs of four to five contact ferrets, and in the nose swabs of two to four contact ferrets in each of the four groups C–F (Table 2). HRSV replication-competent virus was detected in the throat swabs of three to four contact ferrets, and in the nose swabs of one to three contact ferrets in each of these four groups (Table 2). HRSV was detected from day 4–6 to day 8 post-exposure (dpe) in the positive contact ferrets. HRSV RNA concentrations and viral titers were similar in these four groups based on the areas under the curve. In general, viral titers were higher in the throat swabs than in the nose swabs of the contact ferrets (Figure S1). They tended to reach similar peak values in the throat swabs, yet lower peak values in the nose swabs than those in the donor ferrets (Figure S1). HRSV RNA and replication-competent virus was detected in the lung and or BAL of at least one contact ferret in each of the four groups of contact ferrets. RNA concentrations and viral titers in organ samples and BAL of the contact ferrets are given in Table 1.

Upon microscopic examination, pathological changes were observed in several of the respiratory tissues sampled from contact ferrets. The infected mucosae of, especially, the tracheas and nasal turbinates showed mild inflammation characterized by infiltration of mainly neutrophils within the epithelial lining accompanied by exfoliation of degenerated and/or infected epithelial cells and intraluminal exudation of fibrinous material. Large, bright eosinophilic inclusion bodies composed of viral protein may be present within the cytoplasm of infected epithelial cells (Figure 4). In each group, cells positive for HRSV-antigen were detected by immunohistochemistry in one to three contact ferrets. In group C, positive ciliated epithelial cells were found in the trachea and primary bronchus of one contact ferret. In group D, positive ciliated epithelial cells were found in the nasal turbinates or trachea of three contact ferrets. In group E, positive ciliated epithelial cells were found in the trachea and/or primary bronchus of three contact ferrets, including positive syncytial cells (Figure 4). In group F, positive ciliated epithelial cells were found in the nasal turbinates of one contact ferret.

### 3.3. Virus

A low-passage clinical isolate of HRSV was used to inoculate the donor ferrets. The F and G genes of the inoculum virus, as well as of shed virus sampled from the throat or nose swabs of donor and contact ferrets, were sequenced to evaluate any genetic changes potentially marking adaptation to the ferret model (Table S2). Three substitutions in the HRSV G protein and one substitution in HRSV F protein were detected in four of the contact ferrets. Point mutations resulted in amino acid changes T226I in the G protein in animal #5 (group C), Q81Q/L in the G protein in animal #7 (group C), N20N/D in the G protein in animal #14 (group D), and T244T/A in the F protein in animal #23 (group F). No consistent changes in amino acid sequences were observed, suggesting that transmission of virus is independent of virus adaptation to ferrets.

## 4. Discussion

Immunocompromised ferrets inoculated with HRSV can transmit the virus to contact ferrets that are either immunocompromised or immunocompetent. In general, HRSV viral loads were found similar in immunocompromised and immunocompetent contact ferrets. Although immunocompromised ferrets inoculated intra-nasally tended to have higher RNA or viral loads in the nose swabs than immunocompromised ferrets inoculated intra-tracheally, HRSV was transmitted as efficiently from either group to contact ferrets.

The transmission rate of HRSV in our ferret model using a low-passage HRSV isolate is high, reaching 80 to 100%, based on RT-PCR results. In contrast to the study by Chan et al. [18], we chose to use the immunocompromised ferret model as donors for transmission studies, as it is more relevant for the clinical situation, and in addition more and longer HRSV shedding was observed [16]. It demonstrates the potential of these models to be used for the quantitative assessment of HRSV transmission in the context of intervention studies. The immunocompromised donor ferrets reproduced a prolonged infection with high RNA and viral loads in nose and throat swabs as described previously, confirming the robustness of the model [16]. Ferrets inoculated intra-nasally developed higher RNA and viral loads in the nose than ferrets inoculated intra-tracheally. Ferrets inoculated intra-nasally also tended to have more cells positive for HRSV-antigen in the upper regions of the respiratory tract, i.e., in the nasal turbinates and trachea than ferrets inoculated intra-tracheally. Conversely, ferrets inoculated intra-tracheally tended to have more positive cells for HRSV-antigen deeper down the respiratory tract, in the bronchi and bronchioles, than ferrets inoculated intra-nasally. This supports the fact that the route of transmission can have an impact on HRSV kinetics upon infection, as previously shown for influenza A virus in ferrets [21,22].

Both immunocompromised and immunocompetent contact ferrets effectively developed a productive infection following contact transmission of HRSV. In both donor and contact immunocompromised and immunocompetent ferrets, ciliated epithelial cells along the respiratory tract, from the nasal turbinates to the bronchi and bronchioles, were found infected, in association with inflammatory lesions. Only a few substitutions were sporadically detected in the F and G proteins of HRSV shed by contact ferrets, suggesting that little adaptation to the ferret model had occurred during the prolonged infection in donor immunocompromised ferrets and upon contact transmission. These observations further support the appropriateness of the ferret model to reproduce HRSV infection and pathogenesis as seen in humans, including after contact transmission of the virus. The use of a contact transmission model nonetheless precludes the assessment of the respective role of large droplets and aerosols in the transmission of HRSV. However, and despite differences in RNA and viral loads in the nose, donor ferrets inoculated intra-nasally and donor ferrets inoculated intra-tracheally transmitted HRSV to contact ferrets with similar efficiency. This suggests that HRSV transmission in these ferret models does not correlate with viral loads in the upper respiratory tract. Future studies using molecularly cloned HRSV constructs expressing a reporter gene, like EGFP, may facilitate studies of transmission mechanisms of HRSV.

In conclusion, the ferret models presented in this paper can be used to mimic nosocomial transmission of HRSV in vulnerable patients, as well as population-based transmission in humans, and also can be used to evaluate intervention strategies for the prevention of HRSV transmission.

## Figures and Tables

**Table 1:** HRSV viral titers (log10 TCID_50_/g) and RNA concentrations (log10 virus particles (vp)/mL) in sampled tissues of the respiratory tract of donor and contact ferrets.

Ferret		Viral Titers (log10 TCID_50_/g)					RT-PCR (log10 vp/mL)	
Group	Number	Nasal Turbinates	Trachea	Primary Bronchus	Lung	BAL	Lung	BAL
A.	1	4.9	2.1	-	2.9	5	8.8	7.3
	2	-	-	-	-	-	6.9	5.2
B.	3	6.1	1.7	2.3	4.2	6.8	7.5	7.1
	4	-	-	-	-	-	5.8	3.2
C.	5	5.3	1.4	-	-	2.8	5.6	2.9
	6	-	3.3	-	-	4.3	6.5	5.8
	7	4.3	3	-	2.9	4.5	6	5.5
	8	-	-	-	-	-	-	-
	9	6.1	1.7	-	-	-	NQ	NQ
D.	15	-	1.5	-	2.3	-	6.4	4.3
	16	5	-	-	-	1.8	5.3	4.3
	17	-	-	-	-	-	-	-
	18	-	-	-	-	-	NQ	3.9
	19	3.4	-	-	-	-	-	-
E.	10	6.7	3.5	-	2.4	-	6.5	4.4
	11	6.5	5.2	-	3.2	3.5	6.6	5.5
	12	6.2	5.3	-	3.1	5.8	6.5	6.4
	13	4.6	2.3	-	-	1.8	5.6	NQ
	14	-	-	-	-	-	5.7	NQ
F.	20	-	-	-	-	-	NQ	-
	21	1.4	-	-	-	-	-	-
	22	3.7	-	-	-	-	NQ	-
	23	-	-	-	-	-	NQ	NQ
	24	-	-	-	2.2	-	5.3	NQ

NQ: non quantifiable *C*t values.

**Table 2:** Number of ferrets with throat and nose swabs positive for HRSV RNA and for replication-competent HRSV.

Ferret Group	Throat Swab RT-PCR	Nose Swab RT-PCR	Throat Swab VI	Nose Swab VI
A (*N* = 2)	2	2	2	2
B (*N* = 2)	2	2	2	2
C (*N* = 5)	4	3	4	2
D (*N* = 5)	4	4	4	3
E (*N* = 5)	4	2	4	2
F (*N* = 5)	5	4	3	1

## Supplementary Materials

The following are available online at www.mdpi.com/1999-4915/10/1/18/s1, Figure S1: HRSV virus titer in throat and nose swabs of donor and contact ferrets; Group A: donor ferrets inoculated intra-tracheally; Group B: donor ferrets inoculated intra-nasally; Group C: immunocompromised ferrets in contact with group A ferrets; Group D: immunocompetent ferrets in contact with group A ferrets; Group E: immunocompromised ferrets in contact with group B ferrets; Group F: immunocompetent ferrets in contact with group B ferrets; each line represents a single ferret; Table S1: RT-PCR and sequencing primers and probes; Table S2: Sequence mutations in the F and G genes of virus shed from donor and contact ferrets compared to the inoculum virus.
