# Supplementary material for: Transmission of Human Respiratory Syncytial Virus in the Immunocompromised Ferret Model
Source: Viruses. 2018 Jan 2;10(1):18. doi: 10.3390/v10010018 (PMC5795431; doi:10.3390/v10010018)
Supplement: Supplementary file 1 [file viruses-10-00018-s001.pdf]

Article

Transmission of human respiratory syncytial virus in the ferret model

Leon de Waal [1], Saskia L. Smits [1], Edwin J. B. Veldhuis Kroeze [1,2], Geert van Amerongen [1], Marie O. Pohl [1], Albert D. M. E. Osterhaus [1,3], Koert J. Stittelaar [1,*]

[1] Viroclinics Biosciences BV, Rotterdam 3029AK, The Netherlands; dewaal@viroclinics.com (L.d.W.); smits@viroclinics.com (S.L.S.); edwinvk@gmail.com (E.J.B.V.K.); amerongen@viroclinics.com (G.v.A.); pohl@viroclinics.com (M.O.P.); albert.osterhaus@tiho-hannover.de (A.D.M.E.O.)

[2] Department of Viroscience, Erasmus MC, Rotterdam 3015 CN, The Netherlands

[3] Research Centre for Emerging Infections and Zoonoses, University of Veterinary Medicine, Hannover 30559, Germany

* Correspondence: stittelaar@viroclinics.com; Tel.: +31-88-668-4727

Supplementary Materials:

Figure S1
Table S1
Table S2

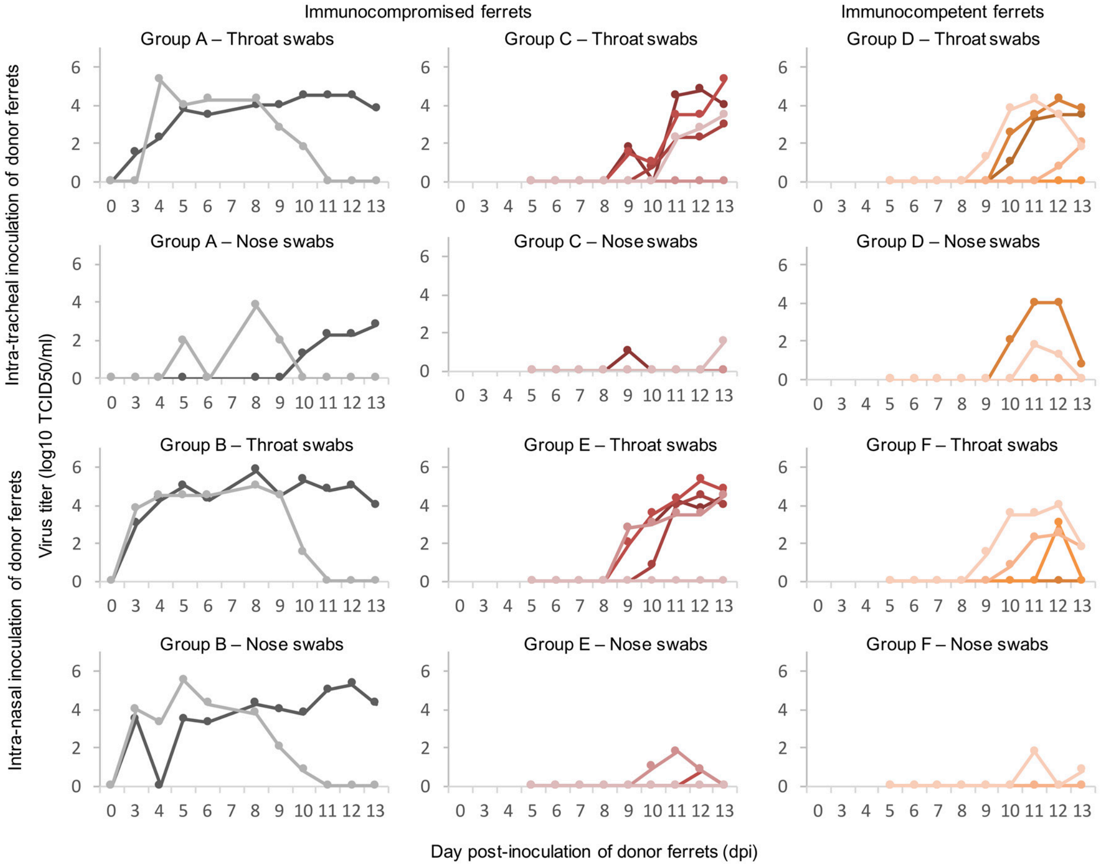

Figure S1. HRSV virus titer in throat and nose swabs of donor and contact ferrets; Group A: donor ferrets inoculated intra-tracheally; Group B: donor ferrets inoculated intra-nasally; Group C: immunocompromised ferrets in contact with group A ferrets; Group D: immunocompetent ferrets in contact with group A ferrets; Group E: immunocompromised ferrets in contact with group B ferrets; Group F: immunocompetent ferrets in contact with group B ferrets; each line represents a single ferret.

Table S1. RT-PCR and sequencing primers and probes

Assay	Virus	Name	Sequence (5'- 3')
qPCR	RSV-A	RSV-A Fwd	AGATCAACTTCTGTCATCCAGCAA
qPCR	RSV-A	RSV-A Rev	TTCTGCACATCATAATTAGGAGTATCAAT
qPCR	RSV-A	RSV-A Probe	Drfly-CACCATCCAACGGAGCACAGGAGAT-BHQ2
qPCR	RSV-B	RSV-B Fwd	TTCCTAACTTCTCAAGTGTGGTCCTA
qPCR	RSV-B	RSV-B Rev	CTGGTTTCTTGGCGTACCTCTATAC
qPCR	RSV-B	RSV-B Probe	FAM-TCCCATTATGCCTAGACCTGCTGCATTG-BHQ1
Sequencing	RSV-A	G RT	ATCTCCATCATGATTGCAATACTAAAC
Sequencing	RSV-A	G Fw1	TGGGGCAAATGCAAACATG
Sequencing	RSV-A	G Rev2	GTTGATTGATAAAATTCTTCAGTGATG
Sequencing	RSV-A	G Rev 1	GCTGCATATGCTGCAGGGTAC
Sequencing	RSV-A	G Fw 2	CACCACAAAACAACGCCAAAA
Sequencing	RSV-A	RSV-AFcDNA	AGCATATGCAGCAACAATCCAAC
Sequencing	RSV-A	RSV-AFFw4	CAAGAACCGACAGAGGATGG
Sequencing	RSV-A	RSV-AFOuterFw	CCATGACCAAATCAAACAGAATC
Sequencing	RSV-A	RSV-AFRev3	GCATGACACAATGGCTCCTA
Sequencing	RSV-A	RSV-AFOuterRev	AATTATGACTAAAATGACACCTCTTACC
Sequencing	RSV-A	RSV-AFFw1	AAAATCAACTCTGGGGCAAATAAC
Sequencing	RSV-A	RSV-AFRev 1	GTGCAGGACCTTGGATACGG
Sequencing	RSV-A	RSV-AFFw2	GTCGAGCCAGAAGAGAACTACC
Sequencing	RSV-A	RSV-AFRev2	AGTAACTTTGCTGTCTAACTATTTGAAC
Sequencing	RSV-A	RSV-AFFw3	AGAACAACAGACTACTAGAGATTACCAG
Sequencing	RSV-A	RSV-AFRev4	TGGATTTACCAGCATTTACATTATG
Sequencing	RSV-A	RSV-AFFw5	AAAGGTGAACCAATAATAAATTTCTATG
Sequencing	RSV-A	RSV-AFRev5	TTACCATTCAAGCAATGACCTCG

Table S2. Sequence mutations in the F and G genes of virus shed from donor and contact ferrets compared to the inoculum virus.

Ferret Group	Ferret Number	Sample type	Dpi	Ct value	F gene	G gene
A	1	Throat swab	5	24.84		
A	1	Nose swab	5	37.15	ND	ND
A	1	Throat swab	11	20.07		
A	2	Throat swab	5	18.45		
A	2	Nose swab	5	32.30	ND	
A	2	Throat swab	11	20.05		
B	3	Throat swab	5	20.40	ND	
B	3	Nose swab	5	22.51	ND	
B	3	Throat swab	11	18.48	ND	
B	4	Throat swab	5	23.34		
B	4	Nose swab	5	17.90	ND	
B	4	Throat swab	11	26.67		
C	5	Throat swab	11	21.36		T226I
C	6	Throat swab	11	27.01		
C	7	Throat swab	11	22.42		Q81Q/L
C	8	Throat swab	11	31.70	ND	
C	9	Throat swab	11	29.56		
D	10	Throat swab	11	19.08		
D	11	Throat swab	11	25.04		
D	12	Throat swab	11	23.55		
D	13	Throat swab	11	25.32		
D	14	Throat swab	11	33.10	ND	Partial; N20N/D
E	15	Throat swab	11	24.40		
E	16	Throat swab	11	24.03		
E	17	Throat swab	11	33.58	ND	Partial
E	18	Throat swab	11	29.40	ND	
E	19	Throat swab	11	22.33		
F	20	Throat swab	11	32.87	ND	Partial
F	21	Throat swab	11	33.03	ND	Partial
F	22	Throat swab	11	30.68	ND	
F	23	Throat swab	11	29.50	T244T/A	
F	24	Throat swab	11	23.91		

Dpi: day post-inoculation; ND: no data
